# Chemical Profile and Bioactivity of *Rubus idaeus* L. Fruits Grown in Conventional and Aeroponic Systems

**DOI:** 10.3390/plants13081115

**Published:** 2024-04-16

**Authors:** Chiara La Torre, Monica Rosa Loizzo, Luca Frattaruolo, Pierluigi Plastina, Antonio Grisolia, Biagio Armentano, Maria Stella Cappello, Anna Rita Cappello, Rosa Tundis

**Affiliations:** 1 Department of Pharmacy, Health and Nutritional Sciences, University of Calabria, 87036 Rende, Italy; chiara.latorre@unical.it (C.L.T.); monica_rosa.loizzo@unical.it (M.R.L.); luca.frattaruolo@unical.it (L.F.); pierluigi.plastina@unical.it (P.P.); annarita.cappello@unical.it (A.R.C.); 2 Azienda Agricola Grisolia A., Contrada Campotenese sn, 87016 Morano Calabro, Italy; grisoliantonio@gmail.com; 3 Azienda Agricola Armentano F., Contrada Campotenese, n. 64, 87016 Morano Calabro, Italy; biagio30@live.it; 4 Institute of Science of Food Production (ISPA), Italian National Research Council, 73100 Lecce, Italy; maristella.cappello@ispa.cnr.it

**Keywords:** α-glucosidase, aeroponic, antioxidant activity, HPLC-DAD, nitric oxide, pancreatic lipase, raspberry

## Abstract

Raspberry (*Rubus idaeus* L.) is a fruit of great interest due to its aroma, nutritional properties, and the presence of many bioactive compounds. However, differences among cultivation systems can affect its composition and, consequently, its potential bioactivity. Herein, for the first time, raspberries grown in an aeroponic system were investigated for their chemical profile and antioxidant and anti-inflammatory activity, as well as their enzyme (α-glucosidase and pancreatic lipase) inhibitory properties in comparison to wild and conventionally cultivated fruits. High-performance liquid chromatography coupled with diode array detection (HPLC-DAD) analyses revealed the presence of gallic acid, caffeic acid, chlorogenic acid, *p*-coumaric acid, ferulic acid, rutin, and catechin in all the samples. The extracts exhibited in vitro anti-inflammatory activity (inhibition of nitric oxide production) regardless of the cultivation method. Of particular interest is the ability of raspberries to inhibit pancreatic lipase. With the exception of the β-carotene bleaching test, the raspberries grown in conventional and aeroponic systems were more active in terms of antioxidants than wild fruits, as evidenced by the ABTS (IC_50_ in the range 1.6–3.4 μg/mL), DPPH (IC_50_ in the range 8.9–28.3 μg/mL), and FRAP tests (24.6–44.9 μM Fe(II)/g). The raspberries from aeroponic cultivation were generally able to exert the same bioactivity as those obtained from both conventionally cultivated and wild fruits, supporting the consideration that in the future, this technology could reshape agriculture by mitigating resource constraints, fostering sustainable practices and increasing yields.

## 1. Introduction

Oxidative stress plays an important role during the development of several chronic degenerative diseases, including diabetes, obesity, cancer, and neurodegenerative diseases [1,2]. The human body uses several mechanisms to minimize the damage induced by the excessive production of free radicals, among which an important role is played by antioxidants. In recent years, great interest has been paid to antioxidants from natural sources, particularly phenolic compounds [3,4].

Red fruits are among the main natural sources of phenolic compounds [5]. The best-known and consumed red fruits belong to the Ericaceae (blueberry) and the Rosaceae (strawberry, blackberry, and raspberry) families.

Raspberries (*Rubus idaeus* L.) belong to the genus *Rubus* that comprises more than 500 species and thousands of cultivars. Raspberries are a red fruit that is highly distinguished for their characteristic color and flavor; they are used for the production of jams, beverages, bakery goods, jellies, dairy products, such as ice cream and yogurt, and fruit syrups.

Raspberries are a rich source of carbohydrates, dietary fiber, vitamins, minerals, proteins, and several phytochemicals, such as phenolics, carotenoids, tocopherols, and unsaturated fatty acids, which are useful for the prevention of some degenerative diseases [6,7,8,9,10,11,12,13]. 

However, the concentration of phenolics and, in general, secondary metabolites is influenced by different factors, including cultivation, ripeness, soil characteristics, irrigation, post-harvest technologies, and climatic conditions [14,15,16]. 

Hence, there is an increased interest in new technology in farming and agricultural practices to improve efficiency, productivity, and sustainability. This interest is further supported by other dynamics that pose substantial implications for future food production and safety [17,18,19,20,21,22]. In order to partially solve this condition, the Food and Agriculture Organization (FAO) has suggested improving soilless-based agriculture techniques, such as aquaponics, hydroponics, and aeroponics [23]. Aeroponics is a modern technique for growing agricultural plants by providing a nutrient solution in the air without soil. Plant roots receive a nutrient spray mist from an atomizing nozzle [18]. 

Improved food production spaces and water-saving methods under soilless culture have led to promising results around the world [24]. 

Some studies indicate that soilless systems provide superior nutrition compared to traditionally grown produce, whereas other works showed no difference or that soil-based production is higher regarding nutritional parameters [25]. Most studies have focused on leafy greens, lettuce, and tomato. Limited research has been published on the soilless cultivation of red berries, with the majority focusing on the cultivation of strawberries [26,27,28]. Treftz and Omaye compared the differences in bioactive compounds, moisture content, and soluble solids in strawberries and raspberries grown in soil and soilless conditions [25]. Their study demonstrated that the content of bioactive compounds from soilless-grown raspberries was equal to or even higher than that of soil-grown fruits, re-iterating the concept that nutrient density in plants grown with soilless systems probably also depends on environmental conditions, such as water stress, the bioavailability of fertilizers, and the cultivar of interest. In a recent study, the modification of the substrate (coconut fiber), consisting of the use of various organic and mineral additives, was used in the soilless cultivation of raspberries [29]. A significant effect of these additives was observed in the biosynthesis of phenolics in raspberries. The fruits were also characterized by a significantly different profile in terms of these compounds. 

In this context and for the first time, the current study was designed with the aim of investigating the chemical profile and the potential effects of raspberries grown in an aeroponic system to act as antioxidant, anti-inflammatory, and pancreatic lipase inhibitor agents in comparison to conventionally cultivated and wild raspberries.

## 2. Materials and Methods

### 2.1. Chemicals and Reagents

The solvents used in this study were obtained from VWR International s.r.l. (Milan, Italy). Gallic acid, caffeic acid, chlorogenic acid, *p*-coumaric acid, ferulic acid, ellagic acid, quercetin, catechin, rutin, ascorbic acid, propyl gallate, butylated hydroxytoluene (BHT), β-carotene, linoleic acid, pancreatic lipase, Tween 20, sodium potassium tartrate, sodium chloride, sodium carbonate, Folin-Ciocalteu reagent, 2,2-diphenyl-1-picrylhydrazyl (DPPH), 2,4,6-tripyridyl-s-triazine (TPTZ), *o*-dianisidine (DIAN) color reagent, peroxidase-glucose oxidase (PGO), 2,2′-azino-bis(3-ethylbenzothiazoline-6-sulfonic acid) diammonium salt, (ABTS) solution, sodium acetate, β-carotene, linoleic acid, 3-(4,5-dimethyl-2-thiazolyl)-2,5-diphenyl-2*H*-tetrazolium bromide (MTT), Dulbecco’s Modified Eagle Medium (DMEM), dimethyl sulfoxide (DMSO), and Fetal Bovine Serum (FBS) were purchased from Sigma-Aldrich s.r.l. (Milan, Italy). l-Glutamine and penicillin/streptomycin were purchased from Gibco, Life Technologies (Waltham, MA, USA). 

### 2.2. Plant Materials 

Conventionally cultivated (soil-grown) raspberries were harvested from plants of Grisolia farm (Calabria, Italy). 

Wild raspberries were harvested and authenticated by dr. Antonio Grisolia in the area of Pollino National Park (Southern Italy) (WGS84: 39°78′33″ N, 16°03′95″ E).

The raspberries were produced in an aeroponics pilot plant at Grisolia farm and Armentano farm (Calabria, Italy). The aeroponic cultivation system, provided and engineered by Hydro Fields s.r.l. (Morano Calabro, Cosenza, Italy), is composed of structures made of anodized aluminum, onto which a plant containment system, nutrient misting system, and an LED lighting system were mounted. 

Continuous carbon dioxide measurements were performed using VAISALA OYJ (Vantaa, Finland) mod. Indigo 500 with a VAISALA OYJ CARBOCAP GMP 251 (Vantaa, Finland) probe to control CO_2_ concentration in the cultivation environment. The water demineralization system with reverse osmosis is produced by Idrotecnica s.r.l. (Genova, Italy). The UV disinfection system for recirculating the nutrient solution is a SITA-UV 440 (Genova, Italy). The fertigation system consists of an Ebara AM15 pump and a continuous analysis system made up of Chemitec s.r.l. (Firenze, Italy) mod. 50 series, S470 probe, S411 probe, and S401 probe, which were used to measure the NH_4_^+^, NO_3_^−^, K^+^, and Cl^−^ concentrations, as well as the conductivity and pH of the nutrient solution. The LED lighting system is made by Bridgelux mod and consists of EB 40E3000 bars (Fremont, CA, USA), 200 lm/w, and a color temperature of 4000 K.

All the raspberry fruits were harvested at the full maturity stage and transported to the laboratory of the Department of Pharmacy, Health and Nutritional Science, University of Calabria (Italy), where they were immediately subjected to the extraction procedure.

### 2.3. Extraction Procedure

The aeroponic (350 g), conventionally cultivated (350 g), and wild raspberries (350 g) were examined for integrity, washed (to remove superficial contamination), cut into small pieces, and subjected to exhaustive maceration by using ethanol as a solvent (1.2 L × 72 h).

This extraction procedure was repeated four times, and after each extraction cycle, the solutions were filtered, combined, and then evaporated under reduced pressure in order to obtain dry extracts. The extracts were stored in brown glass bottles and kept at 4 °C before chemical and biological analyses.

### 2.4. Bioactive Compounds Analysis of Raspberry Fruits

#### 2.4.1. Determination of Total Phytochemicals (Phenols, Flavonoids, and Anthocyanins)

Total phenolic content (TPC) was determined using the Folin-Ciocalteu method, as previously described [30]. The Folin-Ciocalteu reagent is a mixture of phosphomolybdate and phosphotungstate that, upon reaction with phenols, produces a blue color that absorbs at 765 nm. The raspberry extracts (concentration of 1.5 mg/mL) were mixed with water, sodium carbonate 15% (*w*/*v*), and Folin-Ciocalteu reagent. 

After 2 h of incubation at room temperature, the absorbance was read at 765 nm by using a UV-vis spectrophotometer Jenway 6003 (Milan, Italy). TPC was determined in triplicate and was reported as mg of chlorogenic acid equivalents (CGAEs)/100 g plant material (fresh weight (fw)).

For the determination of total flavonoid content (TFC), the raspberry extract—at a concentration of 1.5 mg/mL—was added to distilled water and sodium nitrite 5% (*w*/*v*). After 5 min, aluminum chloride 10% (*w*/*v*) was added. After another 6 min, 1 M sodium hydroxide and water were added. Then, the absorbance was read at 510 nm by using a UV-vis Jenway 6003 spectrophotometer (Milan, Italy). TFC, determined in triplicate, was reported as mg quercetin equivalents (QEs)/100 g plant material (fw) [30].

Total monomeric anthocyanin content (TAC) was assessed by using the pH-differential method reported by Giusti [31] with slight modifications. In brief, 0.5 mL of the extract at the concentration of 1.5 mg/mL was mixed with (a) 3.5 mL of potassium chloride buffer (0.025 M, pH 1) or (b) 3.5 mL of sodium acetate buffer (0.025 M, pH 4.5). After 15 min, the absorbance of each solution was measured at 510 and 700 nm by using a UV-vis Jenway 6003 spectrophotometer (Milan, Italy). The absorbance difference (A) was calculated as follows: A = [(A_510_ − A_700_) pH 1.0 − (A_510_ − A_700_) pH 4.5]. The total anthocyanin content (TAC) was calculated using the molar absorptivity (ε) and molecular weight (MW) of cyanidin 3-*O*-glucoside (ε = 26,900; MW = 449.2). The results are expressed as milligrams cyanidin 3-*O*-glucoside equivalents/100 g plant material (fw).

#### 2.4.2. High-Performance Liquid Chromatography—Diode Array Detection (HPLC-DAD) Analyses

The ethanol extracts of the raspberry fruits were analyzed using high-performance liquid chromatography with diode-array detection (HPLC-DAD). An aliquot (20 μL) of each raspberry ethanol extract was injected into a Shimadzu (Kyoto, Japan) HPLC system equipped with a diode array detector (SPDM10Avp) according to a reported method with slight modifications [32]. Chromatographic separation was performed on a Mediterranean SEA C_18_ column (4.6 mm i.d. × 25 cm, 5 μm). 

The mobile phase comprised 0.1% formic acid in water (A) and methanol (B). The gradient used was the following: 0.01 min, 3% B; 0.01–5 min, 3→5% B; 5.01–10 min, 5→8% B; 10.01–15 min, 8→13% B; 15.01–19 min, 13→15% B; 19.01–47 min, 13→40% B; 47.01–64 min, 40→65% B, 64.01–66 min, 65→95% B; 66.01–69 min 95% B; 69.01–74 min 95→3% B, and remains at 3% B to restore flow equilibrium to the column. 

The flow rate and column temperature were maintained at 0.7 mL/min and at room temperature, respectively. The HPLC analyses were evaluated in the range of 270–365 nm. Specifically, quantitative analysis for different compounds was conducted as follows: gallic acid at 270 nm; caffeic acid at 273 nm; chlorogenic acid at 327 nm; *p*-coumaric acid at 310 nm; ferulic acid at 325 nm; quercetin at 365 nm; rutin at 360 nm; ellagic acid at 254 nm; catechin at 280 nm. The identification was made by comparing the samples with the retention times and characteristic UV-Vis spectra of the pure, standard compounds. Individual components were analyzed quantitatively by using the external standard method and following the pure compounds: gallic acid, caffeic acid, chlorogenic acid, *p*-coumaric acid, ferulic acid, ellagic acid, quercetin, catechin, and rutin. The calibration curves for the standards were prepared according to five appropriate concentrations. 

### 2.5. In Vitro Antioxidant Activity 

The antioxidant properties of the raspberry extracts were explored by using a multi-target approach to test their ability to act according to different mechanisms of action. 

In this study the following assays were performed in vitro: 2,2-azino-bis(3-ethylbenzothiazoline-6-sulfonic acid) (ABTS) test, 1,1-diphenyl-2-picrylhydrazyl (DPPH) test, Ferric Reducing Ability Power (FRAP) test, and β-carotene bleaching test.

In the ABTS test, the ABTS^·+^ radical cation solution was prepared by mixing the ABTS solution (7 mM) and potassium persulphate (2.45 mM), as previously reported [33]. After 12 h, this solution was diluted with ethanol to a final absorbance at 734 nm of 0.70. Successively, 2 mL of diluted ABTS solution was added to the extract (25 μL) at concentrations in the range 1–400 μg/mL. After 6 min, the absorbance was read at 734 nm by using the UV-Vis Jenway 6003 spectrophotometer (Carlo Erba, Milan, Italy). Ascorbic acid was used as a positive control, and the results are expressed as IC_50_ (half maximal inhibitory concentration) values (μg/mL).

The DPPH test was performed as previously described [33]. In brief, the DPPH solution (1.0 × 10^−4^ M) and the raspberry extracts at concentrations in the range of 62.5–1000 μg/mL were mixed. After 30 min, the absorbance was read at 517 nm. Ascorbic acid was used as a positive control. The results are reported as IC_50_ values (μg/mL).

The ability of raspberries to reduce iron ions was assessed by using FRAP tests [33]. A solution of tripyridyltriazine (TPTZ, 10 mM), 2.5 mL of FeCl_30_ (20 mM), HCl (40 mM), and 25 mL of acetate buffer (0.3 M) at pH 3.6 was prepared in order to obtain the FRAP reagent.

This reagent (2.0 mL) was mixed with water (900 μL) and the extracts (at the concentration of 1.25 mg/mL). After 30 min of incubation, the absorbance was measured at 595 nm by using the UV-Vis Jenway 6003 spectrophotometer. Butylated hydroxytoluene (BHT) was used as a positive control. The FRAP value is expressed as μM Fe(II)/g. 

The β-carotene bleaching test was applied to evaluate the ability of the raspberry extracts to protect lipid peroxidation [33]. In brief, a mixture of β-carotene, linoleic acid, and 100% Tween 20 was prepared. The obtained emulsion was added to a 96-well microplate containing samples at concentrations ranging from 100 to 2.5 μg/mL. The absorbance was measured at 470 nm by using the UV-Vis Jenway 6003 spectrophotometer against a blank at t = 0 and after 30 and 60 min of incubation. Antioxidant activity (AA) was calculated by using the following equation:

AA = [(A_0_ − A_t_)/(A_0_* − A_t_*)] × 100

where A_0_ and A_0_* are the absorbance values obtained at time 0 for the samples and control, respectively, while A_t_ and A_t_* are the absorbance values obtained after 30 and 60 min of incubation for the samples and controls, respectively. Propyl gallate was a positive control. The results are reported as IC_50_ values (μg/mL).

### 2.6. Anti-Inflammatory Activity

#### 2.6.1. Cell Cultures

The RAW 264.7 murine macrophage cell line was purchased from the American Culture Collection (ATCC, Manassas, VA, USA) and cultured in DMEM (Sigma, St. Louis, MO, USA) supplemented with 10% Fetal Bovine Serum (FBS), 2 mM l-glutamine, and 1% penicillin/streptomycin. The cells were cultured at 37 °C in a humidified atmosphere with 5% CO_2_ [34]. 

#### 2.6.2. Inhibition of NO Production in LPS-Stimulated RAW 264.7 Cells

Griess reagent was used to determine the presence of nitrites, the stable oxidized products of NO in cell culture media [35]. The extracts were solubilized in dimethyl sulfoxide (DMSO), and the solvent was kept at less than 1%. The RAW 264.7 cells were seeded in 24-well plates at a density of 2 × 10^5^ cells/well in DMEM. The following day, the cells were simultaneously stimulated with LPS (1 µg/mL) and treated for 24 h with increasing concentrations of extracts (ranging from 10 to 400 µg/mL). DMSO was used as a control vehicle. Finally, 100 µL of cell culture medium was mixed with 100 µL of Griess reagent in a 96-well plate, and the absorbance was measured at 550 nm by using a BioTek Synergy H1 microplate reader (Agilent Technologies, Santa Clara, CA, USA).

### 2.7. Cell Viability Assay

Cell viability was determined by using a 3-(4,5-Dimethyl-2-thiazolyl)-2,5-diphenyl-2H-tetrazolium bromide (MTT, Sigma-Aldrich, St. Louis, MO, USA) assay as previously described [36]. Briefly, the RAW 264.7 cells were treated for 24 h with increasing concentrations of extracts (from 10 to 400 µg/mL). After treatment, MTT solution was added to each well (to a final concentration of 0.5 mg/mL), and the plates were incubated at 37 °C for 2 h until the formation of formazan crystals. DMSO-solubilized formazan in each well was quantified by reading the absorbance at 570 nm using a microplate reader.

### 2.8. α-Glucosidase Inhibitory Activity Assay

In the α-glucosidase inhibition activity test, a maltose solution was prepared by mixing 12 g of maltose in 300 mL of sodium acetate buffer 50 mM [37]. The enzyme solution was prepared by using 1 mg of enzyme in 10 mL of ice-cold distilled water. The *o*-Dianisidine (DIAN) color reagent solution was prepared by dissolving 1 tablet in 25 mL of distilled water. The peroxidase-glucose oxidase (PGO) system-color reagent solution was obtained by dissolving 1 capsule in 100 mL of ice-cold distilled water. The samples (5 μL at a concentration in the range of 12.50–1000 g/mL) and control were stirred into the maltose solution and left to equilibrate at 37 °C. The reaction was started with the addition of a α-glucosidase solution. After 30 min of incubation at 37 °C, the reaction was stopped by adding 50 μL of a solution of perchloric acid prepared by adding 4.2 mL of perchloric acid in 95.8 mL of distilled water. The supernatant of the tube of step one was mixed with DIAN and PGO and was left to incubate at 37 °C for 30 min. The absorbance was read at 540 nm. Acarbose was used as the positive control. The inhibitory activity is expressed as an IC_50_ value (μg/mL).

### 2.9. Pancreatic Lipase Inhibitory Activity Test

Pancreatic lipase is an important enzyme that is responsible for the digestion of dietary fats (hydrolyzes 56% of fats), and its inhibition is the most widely studied mechanism for the identification of new potential anti-obesity agents [37]. Herein, we assessed the pancreatic lipase inhibitory activity of the raspberry extracts by using an assay that we previously described [35]. In brief, the extract (at different concentrations), 4-nitrophenyl octanoate (NPC), Tris-HCl buffer (pH 8.5), and enzyme solution were added to a 96-well plate and incubated at 37 °C for 30 min. The absorbance was read at 405 nm by using the UV-Vis Jenway 6003 spectrophotometer. Orlistat was used as the positive control.

The inhibitory activity is expressed as an IC_50_ value (μg/mL).

### 2.10. Statistical Analysis

The results were reported as the mean ± standard deviation of three independent measurements ± standard deviation (SD). All data were analyzed using a one-way analysis of variance (ANOVA) statistical (Prism GraphPad 4.0 software, GraphPad Inc., San Diego, CA, USA) and Tukey’s post-hoc testing (GraphPad Prism 8 software, GraphPad Inc., San Diego, CA, USA). A *p* value of <0.01 was considered statistically significant. 

## 3. Results and Discussion

### 3.1. Bioactive Compound Content in Raspberry Fruit

The aeroponic, soil-grown (conventionally cultivated), and wild raspberries were subjected to exhaustive maceration using ethanol as the solvent. 

Aliphatic alcohols (e.g., ethanol and methanol) and polar organic solvents (e.g., ethyl acetate and acetone) are the most popular solvents used to extract phenolics from plants [38].

A large part of the extraction procedures includes no generally recognized as safe (GRAS) solvents, which are biologically aggressive and contaminant and are, therefore, inadequate for potential future applications in the pharmaceutical, food, and cosmetic industries [39]. 

The high toxicity of methanol, for example, makes it unfeasible for applications that involve ingestion or contact by humans. 

Instead, ethanol, a solvent that is environmentally friendly and has low toxicity, provides good phenolic extraction and shows a good aptitude for large-scale processes [39].

Both wild and conventionally cultivated raspberries exhibited an extraction yield of 11.2%. A lower yield was obtained for the fruits grown in the aeroponic system (9.2%). 

The wild, conventionally cultivated, and aeroponic system raspberries were analyzed for their total phenolics, flavonoids, and anthocyanins content. As reported in Table 1, the following trend was observed: conventionally cultivated raspberries > raspberries from aeroponic system > wild raspberries.

The raspberries grown in the aeroponic system exhibited intermediate content (between conventionally cultivated and wild fruits). In fact, the conventionally cultivated sample has the highest content of phenolics (501.1 mg equivalents of chlorogenic acid/100 g plant material), flavonoids (26.3 mg equivalents of quercetin/100 g plant material), and anthocyanins (91.6 mg equivalents of cyanidin 3-*O*-glucoside/100 g plant material), followed by the sample from the aeroponic system and the wild sample.

Wu et al. [40] analyzed the TPC and TFC of the methanol (100%), ethanol (100%), 50% ethanol, 50% methanol, ethyl acetate, and acetone extracts from the leaves, fruit pulp, and seeds of raspberries from China; they showed that TPC and TFC significantly depend on the different solvents used for the extraction procedure. Regardless of the plant parts investigated (leaves, pulp, or seed), both hydroalcoholic solutions (50% methanol or ethanol) showed high efficiency in the extraction of phenolic compounds, followed by ethanol (100%) and methanol (100%). This probably depends on the polarity of the compounds present in the samples [41,42]. Raspberry pulp is characterized by the presence of gallic acid, procyanidin C3, and ellagic acid as the most abundant constituents [40].

The phenolics in the ethanol extract of three different raspberry samples (wild, conventionally cultivated, and aeroponic system) (Figure S1, Supplementary Materials) were investigated. The phenolic profile of the three samples was similar, and seven compounds were identified and quantified (Table 2).

The presence of gallic acid, caffeic acid, *p*-coumaric acid, chlorogenic acid, ferulic acid, catechin, and rutin was observed in all the samples, with the exception of ferulic acid, which was not detected in the extract of the aeroponic raspberries.

Rutin (quercetin-3-*O*-rutinoside) and chlorogenic acid were found as the main compounds in all the samples. No differences were observed in the amount of phenolics between the cultivated and wild type raspberries. Interestingly, some differences in the amount of some phenolics were observed in the aeroponics raspberries, with respect to the cultivated and wild type samples.

The amount of rutin in the aeroponics raspberries was significantly higher than that observed in the cultivated and wild type samples (76.5 ± 0.1 vs. 68.1 ± 0.2 and 67.4 ± 0.4 mg/kg, respectively). A similar trend was observed in the case of catechin, the levels of which were higher in the aeroponic samples when compared to the cultivated and wild type samples (50.5 ± 0.5 vs. 40.1 ± 0.1 and 39.6 ± 0.9 mg/kg, respectively). On the other hand, no significant differences among the different samples were observed in the case of gallic acid, chlorogenic acid, caffeic acid, and *p*-coumaric acid.

Bortolini et al. [43] reviewed the studies published in the last decade about red fruits, including raspberries, that evaluate aspects related to their chemical composition, biological activity, and potential technological applications. When analyzing the chemical constituents, which are in agreement with the results obtained in the present study, raspberries were characterized by the presence of, among others, chlorogenic acid, gallic acid, rutin, caffeic acid, and *p*-coumaric acid, and caffeic acid. Moreover, quercetin was not identified by these researchers.

The chemical profile of the methanol (100%), ethanol (100%), 50% ethanol, 50% methanol, ethyl acetate, and acetone extracts of the leaves, fruit pulp, and seeds of raspberries from China was analyzed by Wu et al. [40]. The fruit pulp is characterized by the presence of gallic acid, procyanidin C3, and ellagic acid as the most abundant constituents [40].

### 3.2. Antioxidant Activity

Free radicals play an important role in the development of inflammation status and several degenerative diseases, including diabetes, obesity, and cancer [44]. Many studies have indicated that bioactive compounds serve as the main contributors to the antioxidant potential in various plant extracts and foods. These molecules, which often constitute highly complex mixtures, act through diverse antioxidant mechanisms, including inhibiting lipid oxidation, scavenging free radicals, and reducing or chelating transition metal ions. It is, therefore, important to evaluate antioxidant effects by using assays with different mechanisms of action. In this context, the potential antioxidant properties of the soil-grown, wild, and aeroponic system raspberries were analyzed by using ABTS, DPPH, FRAP, and β-carotene bleaching tests.

The DPPH and ABTS tests were used to assess the radical scavenging activity of raspberries. The DPPH test is based on the reduction of the purple DPPH· radical to 1,1-diphenyl-2-picryl hydrazine, whereas the ABTS test is based on the generation of a blue/green ABTS^·+^ radical cation that can be reduced by an antioxidant agent. The FRAP test involves the reduction of ferric iron (Fe^3+^) to ferrous iron (Fe^2+^), which is monitored while the β-carotene bleaching test is applied to evaluate the ability of raspberry extracts to inhibit lipid peroxidation in both the initiation and propagation phases.

The raspberries exhibited concentration-dependent antioxidant activity in all these assays. The data are reported in Table 3.

In both the DPPH and ABTS tests, the conventionally cultivated (soil-grown) raspberries showed the best activity, with IC_50_ values of 8.9 and 1.6 μg/mL, respectively. This activity could be related to the higher total phenolics, flavonoids, and anthocyanins content of this extract. The radical scavenging activity of the raspberries from aeroponic system are of interest also, with IC_50_ values of 11.5 and 2.9 μg/mL in DPPH and ABTS tests, respectively.

The wild and conventionally cultivated raspberries inhibited lipid peroxidation in the β-carotene bleaching test, with IC_50_ values of 5.6 and 5.1 μg/mL, respectively, after 30 min of incubation, and IC_50_ values of 5.4 and 5.2 μg/mL, respectively, after 60 min of incubation.

The extract of the raspberries grown using an aeroponic system demonstrated approximately four times less antioxidant activity after 30 min of incubation and even less after 60 min of incubation (inhibition of lipid peroxidation of 42.6% at 100 μg/mL).

Table 3 also shows the data obtained by applying the FRAP test. With the exception of wild samples, the other two raspberry extracts (44.9 and 42.4 μM Fe(II)/g for raspberries grown in conventional and aeroponic systems, respectively) exhibited higher activity than BHT, which was used as the positive control (32.44 μM Fe(II)/g).

The obtained results show higher radical scavenging activity against DPPH (with the exception of wild fruits) when compared to those reported by Wu et al. [38]; the 50% methanol extract from raspberry pulp exhibited an IC_50_ value of 18.71 μg/mL in the same assay.

As reported by de Souza et al. [45], raspberry extract protects against lipid peroxidation, with a percentage of 75.19%, exhibiting a value of 6.27 μmol/g fw (fresh weight) in ABTS tests.

DPPH radical scavenging potential was found for *R. idaeus* grown wildly in Serbia, with an IC_50_ value of 294.79 μg/mL [46].

Protection from lipid peroxidation (evaluated by using the β-carotene bleaching test) from *R. discolor* fruits collected from two different wild-growing populations from Serbia and subjected to different solvent (acetone, ethanol, methanol, and water) extraction procedures was studied. The acetone extracts exhibited the most promising activity, with IC_50_ values of 120.3 and 258.4 μg/mL for Belgrado and Cer, respectively. However, these values are about five times higher than those found for the samples analyzed in the present study [47]. At the same time, the raspberry extracts investigated in this study exhibited a stronger ferric-reducing power than that found by Chwil et al. [48], who investigated the antioxidant potential of the extracts from fruits of three cultivars, such as *R. idaeus* ‘Glen Ample’, ‘Laszka’, and ‘Radziejowa’ (cultivated in Poland); they found a reducing power similar to the cultivar *R. idaeus* ‘Heritage’, *R. innominatus*, and *R. niveus*, but it was lower than *R. caucasicus* × *Chester*, *R. cyri*, and *R. insularis* [49,50].

In the present study, rutin, chlorogenic acid, and gallic acid were the major constituents of the raspberry extracts. Many studies have demonstrated that these phenolics possess strong antioxidant activity [51,52,53].

Zheng et al. [51] reported IC_50_ values of 4.9 and 6.9 μM for rutin and chlorogenic acid, respectively, investigating 38 phenolics for their DPPH radical-scavenging activities. Moreover, rutin showed effective inhibition of lipid peroxidation [52]. A moderate ABTS^·+^ radical scavenging potential was found for this flavonoid by Rusmana et al. [53], with a percentage of inhibition of ABTS^·+^ radical cations of 29.08% at 10 μg/mL. Gallic acid (3,4,5-trihydroxybenzoic acid) exhibited an IC_50_ value of 47.7 μM in DPPH tests [54].

The structure–activity relationship (SAR) analysis, examining 38 phenolics, revealed that an *o*-dihydroxyl structure or 3′,4′-dihydroxyl for flavonoids is the major factor for the remarkable radical scavenging activity [51]. The presence of a 3-hydroxyl group may contribute to increasing anti-radical activity. The hydrogen bonds between the semiquinone group and the *o*-hydroxyl group, the formation of a stable product in the anti-radical reaction, the presence of 3-hydroxyl group and 4-carbonyl group, and the formation of a whole conjugated system in the molecule all contribute to a more effective scavenger. Moreover, the distribution and delocalization of electrons caused by other groups influence the activity of phenolics [51].

### 3.3. Anti-Inflammatory Activity

Inflammatory processes are triggered by different stimuli, including bacterial lipopolysaccharide (LPS), which is widely used to trigger innate immunity through phagocytic cells activation comprising macrophages and propagate the inflammatory process through nitric oxide (NO) production.

NO is one of main mediators of these processes, playing a central role in the modulation of the release of several inflammatory agents [55,56]. Over the years, several reports have demonstrated that natural products from plants, including red raspberries, are able to combat various disorders, such as inflammation [57,58,59].

Here, we evaluated whether aeroponic cultivation could interfere with the anti-inflammatory ability of red raspberries. For this purpose, the ability to inhibit NO production in the raspberry extracts grown in aeroponic conditions was compared with that of the extracts from wild fruits and conventionally cultivated fruits.

**Figure 1:**
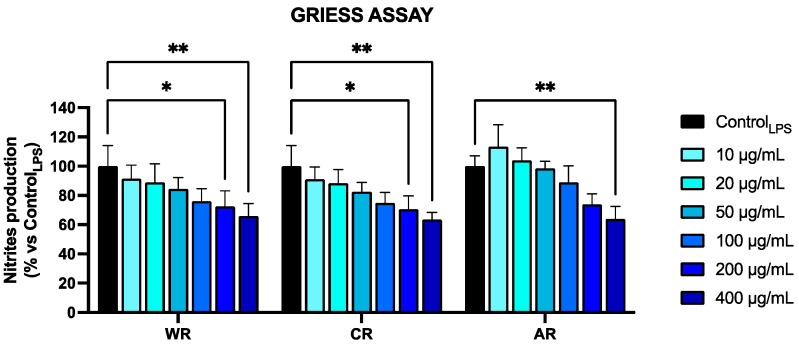
Nitrites levels, measured by Griess assay in the culture medium of RAW 264.7 cells, stimulated for 24 h with LPS and simultaneously treated with different concentrations of raspberry extracts (as indicated). The results are expressed as a percentage of nitrite compared to the control (Control_LPS_). WR: Wild raspberry; CR: Conventionally cultivated raspberry; AR: Raspberries grown in aeroponic conditions. Values represent the mean ± S.D. of three independent experiments, each performed with triplicate samples. * *p* value < 0.05; ** *p* value < 0.01.

The anti-inflammatory activity of red raspberry extracts was tested using Griess assays on RAW 264.7 cells, which is a validated experimental model for this purpose [36,60]. The results obtained, reported in Figure 1, highlight the promising anti-inflammatory activity of the tested extracts.

Specifically, the amount of nitrites detected after treatment with the maximum concentration of the extracts exhibited a statistically significant decrease, equal to 30%, independent of the growing method used.

**Figure 2:**
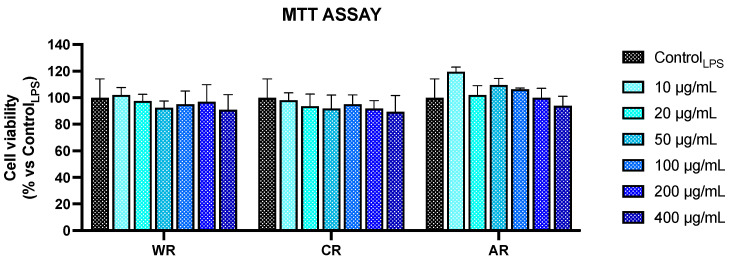
Cell viability, measured by using MTT assays of RAW 264.7 cells stimulated for 24 h with LPS and simultaneously treated with different concentrations of raspberry extracts (as indicated). The results are expressed as percentages of viable, treated cells compared to the control cells (Control_LPS_). WR: Wild raspberries; CR: Conventionally cultivated raspberry; AR: Raspberries grown in aeroponics conditions. Values represent the mean ± S.D. of three independent experiments, each performed with triplicate samples.

Finally, in order to prove that the assessed nitrite reduction was not related to the cytotoxic effects of the extracts, after treatment, cell viability of the RAW 264.7 cells was evaluated using MTT assays. As it is shown in Figure 2, the outcome highlights a lack of cytotoxic effects in the experimental conditions (relative to all the investigated extracts).

### 3.4. Enzymes Inhibition Assay of Raspberry Fruits

The prevalence of obesity and diabetes is increasing and has become a global problem. Obese individuals develop insulin resistance, which is characterized by impaired insulin action in the liver and reduced glucose uptake in fat and muscle [61].

α-Glucosidase inhibitors are a class of agents used for the treatment of type 2 diabetes, which are used alone or in combination with other anti-diabetic drugs [62]. The α-glucosidase inhibitor acarbose has been shown to increase life expectancy in patients affected by type 2 diabetes mellitus and reduce the risk of the development of cardiovascular events in patients with impaired glucose tolerance.

In recent years, pancreatic lipase inhibitors have received considerable attention from researchers regarding treating obesity, and the inhibitors of this enzyme taken from natural products have attracted much attention due to their structural diversity, low toxic effects, and wide range of sources [63]. At present, the only pancreatic lipase inhibitor approved in Europe is Orlistat, the saturated derivative of lipstatin isolated from *Streptomyces toxytricini*. However, its use is compromised by gastrointestinal adverse reactions.

Herein, the impact of the extracts of raspberry fruits on α-glucosidase and pancreatic lipase activity was estimated. The data (IC_50_ values) are reported in Table 4.

The extracts of the wild and conventionally cultivated (soil-grown) fruits (IC_50_ of 5.4 and 5.1 μg/mL, respectively) exhibited the highest inhibitory potential on the enzyme when compared to the extract from the aeroponic system (IC_50_ of 6.8 μg/mL).

A lower activity against pancreatic lipase was found by Fabroni et al. [64] for *R. fruticosus* extract, with an IC_50_ value of 1.99 mg/mL, whereas IC_50_ values of 4.45 and 3.97 mg/mL were recorded for the *R. grandifolius* Funchal and Machico varieties, respectively [65]. Moreover, *R. caesius* extract inhibited lipase, with an IC_50_ value of 9.38 mg/mL [66]. The results obtained from *R. ulmifolius* hydroalcoholic- and anthocyanin-rich extracts were of the same order of magnitude, with IC_50_ values of 67.98 and 23.37 μg/mL, respectively [67]. A similar grade of potency was also observed for *R. sanctus*, with IC_50_ values in the range 6.03–10.31 μg/mL for acetone and aqueous extracts, respectively [68].

The promising lipase inhibitory activity of raspberry extracts could be reasonably caused by the presence of some of the identified phenolics from previous studies [69,70,71,72,73], which exhibit potential anti-obesity activity.

A strong inhibitory effect of raspberry ethanol extracts on pancreatic lipase activity was found, with IC_50_ values in the range 5.1–6.8 μg/mL. All the extracts were more active than the positive control Orlistat (IC_50_ value of 37.1 μg/mL).

Numerous isolated phenolics have been analyzed as potential lipase inhibitors, and the compounds from the raspberry extracts are among these.

Martinez-Gonzalez et al. [70] reported IC_50_ values of 401.5 and 170.2 μM for caffeic acid and *p*-coumaric acid, respectively. IC_50_ values of 3.81 mM for chlorogenic acid, 1.83 mM for catechin, and 5.05 mM for ferulic acid and gallic acid were obtained by Tan et al. [71]. More recently, an IC_50_ value of 367 μM was found for rutin [72].

Karamać et al. [73] investigated different hydroxycinnamic acids as potential anti-obesity agents. Caffeic acid and ferulic acid exhibited the highest pancreatic lipase inhibitory activity. By analyzing the structure–activity relationship, hydroxybenzoic acids less powerfully inhibit the activity of pancreatic lipase when compared to hydroxycinnamic acids, and compounds with a methoxy group are weak inhibitors of the enzyme.

Moreover, the position of hydroxyl groups in the ring affects lipase inhibition. In fact, compounds with hydroxyl groups in the *o*-position were stronger inhibitors than compounds with hydroxyl groups in the *p*-position.

The inhibitory effects of flavonoids generally depend on the number and position of phenolic hydroxyl groups. A high number of hydroxyl groups increases the inhibitory effects [74]. The presence of galloyl moieties and the polymerization of flavan-3-ols enhance pancreatic lipase inhibition. Non-esterified flavan-3-ols, such as (+)-catechin, display a lower inhibitory activity than flavan-3-ol esters. Moreover, higher inhibitory activity was observed after the elimination of glycosylation from both flavonoids and anthocyanins.

When analyzing the ability of raspberries to inhibit α-glucosidase, IC_50_ values in the range 128.44–166.09 μg/mL were found. All the investigated extracts were less active than the positive control acarbose (IC_50_ of 36.2 mg/mL). The sample obtained via the maceration of wild fruits was the most active extract, followed by the conventional cultivated sample and then the aeroponic system sample.

Moreover, in this case, the observed inhibitory effects of the extracts of raspberry fruits might be a synergic effect from the total bioactive compounds present in the extracts.

The effective inhibition of α-glucosidase by the extracts of other red berry fruits was proven. In a previous study, interesting results were obtained from fresh *Arbutus unedo* fruits that were hydro-alcoholic extracted when compared to α-glucosidase, with an IC_50_ value of 28.42 μg/mL, followed by ethanol ultrasound-assisted extraction (IC_50_ value of 40.25 μg/mL) [30].

The ethanol Soxhlet extraction of fresh and dried *Cornus mas* fruits from Italy was able to inhibit α-glucosidase, with IC_50_ values of 30.40 and 54.52 μg/mL, respectively [35].

Previously, Dzydzan et al. [74] found an IC_50_ value of 25.68 μg/mL for *C. mas* red fruits from Poland.

The chemical analysis of samples investigated in this work reveals that raspberry extracts contain several phenolic acids. Simple phenolic acids have been studied for their potential activity and have been shown to increase glucose uptake and glycogen synthesis, improving the glucose and lipid profiles of patients affected by certain diseases such as obesity and diabetes mellitus [75]. SAR analysis revealed that hydroxyl groups might play an important role in α-glucosidase inhibition, too. Phenolic acids that have more than one hydroxyl group, such as caffeic acid, show higher inhibition effects than some phenolic acids with only one hydroxyl group or one hydroxyl group plus one or two methoxy groups in their structure, such as *p*-coumaric acid.

The flavonoid rutin, which characterized all our raspberry extracts, showed stronger inhibition of α-glucosidase, with an IC_50_ value of 0.037 μM [76].

## 4. Conclusions

In this work, raspberries grown using an aeroponic system were investigated for their chemical profile and bioactivity, and this was compared with wild and conventionally cultivated raspberries. Our results prove some differences in the chemical compositions between raspberries grown with nutrient solution and those grown conventionally in soil. Despite the lower extraction yield, the chemical analyses revealed that the raspberries from the aeroponic system exhibited the highest content of rutin and catechin. In contrast, a similar trend was observed for all the extracts in terms of antioxidant, anti-inflammatory, and enzyme-inhibitory activity, regardless of the cultivation method. Of particular interest is the ability of raspberry fruits to inhibit pancreatic lipase when compared to Orlistat. Until now, Orlistat is the only pancreatic lipase enzyme inhibitor available in clinics, and identifying safe alternatives from plants to inhibit lipase is considered to represent a significant advancement.

In summary, this study reveals that raspberries grown in aeroponic systems show comparable biological properties to those grown in soil, supporting the findings that aeroponic technology can reshape modern agriculture by mitigating resource constraints, fostering sustainable practices, increasing yields, and assisting in achieving the zero-hunger sustainable goal.

## Figures and Tables

**Table 1:** Phytochemicals content (TPC, TFC, and TAC) of wild (W), conventionally cultivated (C), and aeroponic system (A) raspberry extracts.

Extract	TPC	TFC	TAC
	(mg CGAE/100 g)	(mg QE/100 g)	(mg CyE/100 g)
Wild	430.5 ± 3.6 ^c^	19.5 ± 0.9 ^b^	85.5 ± 2.2 ^b^
Conventionally cultivated	501.1 ± 4.4 ^a^	26.3 ± 1.0 ^a^	91.6 ± 2.4 ^a^
Aeroponic system	482.7 ± 3.7 ^b^	23.6 ± 1.4 ^a^	86.1 ± 3.4 ^b^
*Sign.*	**	**	**

Data are expressed as mean ± standard deviation (*n* = 3). TPC: Total Phenols Content. TFC: Total Flavonoids Content. TAC: Total Anthocyanins Content. CGAE: chlorogenic acid equivalents. QEs: quercetin equivalents. CyE: Cyanidin 3-*O*-glucoside equivalents. The results followed by different letters are highly significantly different at ** *p* ≤ 0.01.

**Table 2:** The main constituents (mg/kg) of the raspberry ethanol extracts identified using HPLC-DAD.

Compound	Raspberries			*Sign.*
	Wild	Conventionally Cultivated	Aeroponic System	
Caffeic acid	35.8 ± 0.1 ^a^	36.1 ± 1.1 ^a^	35.8 ± 0.1 ^a^	ns
Catechin	39.6 ± 0.9 ^a^	40.0 ± 0.1 ^a^	50.5 ± 0.5 ^b^	**
Chlorogenic acid	65.4 ± 0.2 ^a^	65.7 ± 0.6 ^a^	66.0 ± 0.7 ^a^	ns
*p*-Coumaric acid	32.8 ± 0.1 ^a^	32.8 ± 0.1 ^a^	33.2 ± 0.1 ^a^	ns
Ferulic acid	36.9 ± 0.4 ^a^	37.14 ± 0.7 ^a^	nd	ns
Gallic acid	58.2 ± 1.3 ^a^	59.3 ± 1.2 ^a^	56.6 ± 1.5 ^a^	ns
Quercetin-3-*O*-rutinoside	67.4 ± 0.4 ^a^	68.1 ± 0.2 ^a^	76.5 ± 0.1 ^b^	**

Data are expressed as mean ± standard deviation (*n* = 3). nd: not detected. Differences between groups were evaluated by using one-way ANOVA followed by Tukey’s tests. The results followed by different letters are highly significantly different at ** *p* ≤ 0.01; ns: not significant, *p* > 0.05.

**Table 3:** In vitro antioxidant activity of the raspberry extracts.

Extract	DPPH Test (IC_50_ μg/mL)	ABTS Test (IC_50_ μg/mL)	β-Carotene Bleaching ^ Test (IC_50_ μg/mL)		FRAP Test ^§^ (μM Fe(II)/g)
			30 min	60 min	
Wild	28.3 ± 1.3 ^c^	3.4 ± 0.3 ^c^	5.6 ± 1.8 ^a^	5.4 ± 0.7 ^a^	24.6 ± 1.1 ^b^
Conventionally cultivated	8.9 ± 0.8 ^a^	1.6 ± 0.1 ^a^	5.1 ± 1.4 ^a^	5.2 ± 0.2 ^a^	44.9 ± 1.7 ^a^
Aeroponic system	11.5 ± 1.7 ^b^	2.9 ± 0.3 ^b^	21.1 ± 1.2 ^b^	42.6% ^	42.4 ± 1.6 ^a^
*Sign.*	**	**	**	ns	**
*Positive control*					
Ascorbic acid	5.2 ± 0.9	1.1 ± 0.8			
Propyl gallate			1.3 ± 0.5	1.1 ± 0.3	
BHT					32.9 ± 1.8

Data are expressed as mean ± standard deviation (*n* = 3). ^ tested at a concentration of 100 μg/mL. **^§^** tested at a concentration of 1.25 mg/mL. Differences between groups were evaluated by using one-way ANOVA followed by Tukey’s tests. The results followed by different letters are highly significantly different at ** *p* ≤ 0.01. ns: not significant.

**Table 4:** The α-Glucosidase and pancreatic lipase inhibitory activity of the raspberry extracts.

Raspberry Extract	Pancreatic Lipase (IC_50_ μg/mL)	α-Glucosidase (IC_50_ μg/mL)
Wild	5.4 ± 0.8 ^a^	128.44 ± 3.70 ^a^
Conventionally cultivated	5.1 ± 0.9 ^a^	152.65 ± 3.97 ^b^
Aeroponic system	6.8 ± 0.9 ^b^	166.09 ± 4.08 ^c^
*Sign.*	**	**
*Positive control*		
Orlistat	37.1 ± 1.6	
Acarbose		36.2 ± 1.3

Data are expressed as mean ± standard deviation (*n* = 3). Differences between groups were evaluated by using one-way ANOVA followed by Tukey’s tests. Different superscripts in the same column indicate statistically different values (** *p* ≤ 0.01).

## Data Availability

Data are contained within the article and Supplementary Materials.

## Supplementary Materials

The following supporting information can be downloaded at https://www.mdpi.com/article/10.3390/plants13081115/s1: Figure S1. Chromatograms of raspberry extracts in ethanol. A: wild type; B: cultivated; C: aeroponic. Figure S2. Calibration curve of phenolics used as external standards.
