# Supplementary material for: Chemical Profile and Bioactivity of Rubus idaeus L. Fruits Grown in Conventional and Aeroponic Systems
Source: Plants (Basel). 2024 Apr 16;13(8):1115. doi: 10.3390/plants13081115 (PMC11053529; doi:10.3390/plants13081115)
Supplement: Supplementary file 1 [file plants-13-01115-s001.zip › plants-2949579-supplementary.pdf]

Supplementary Materials

Article

Chemical Profile and Bioactivity of *Rubus idaeus* L. Fruits Grown in Conventional and Aeroponic Systems

Chiara La Torre [1], Monica R. Loizzo [1], Luca Frattaruolo [1], Pierluigi Plastina [1], Antonio Grisolia [2], Biagio Armentano [3], Maria Stella Cappello [4], Anna Rita Cappello [1] and Rosa Tundis [1,*]

[1.] Department of Pharmacy, Health and Nutritional Sciences, University of Calabria, 87036 Rende (CS), Italy; rosa.tundis@unical.it (R.T.); monica_rosa.loizzo@unical.it (M.R.L); annarita.cappello@unical.it (A.R.C.); luca.frattaruolo@unical.it (L.F.); chiara.latorre@unical.it (C.L.T.); pierluigi.plastina@unical.it (P.P).
[2.] Azienda Agricola Grisolia A. – Contrada Campotenese sn - 87016 Morano Calabro (CS), Italy; grisoliantonio@gmail.com (A.G.).
[3.] Azienda Agricola Armentano F. - Contrada Campotenese, n. 64 - 87016 Morano Calabro (CS), Italy; biagio30@live.it (B.A.).
[4.] CNR, Institute of Science of Food Production (ISPA), 73100 Lecce, Italy; maristella.cappello@ispa.cnr.it (M.S.C.).

*Correspondence: rosa.tundis@unical.it (R.T.); Tel.: +39-0984-493246.

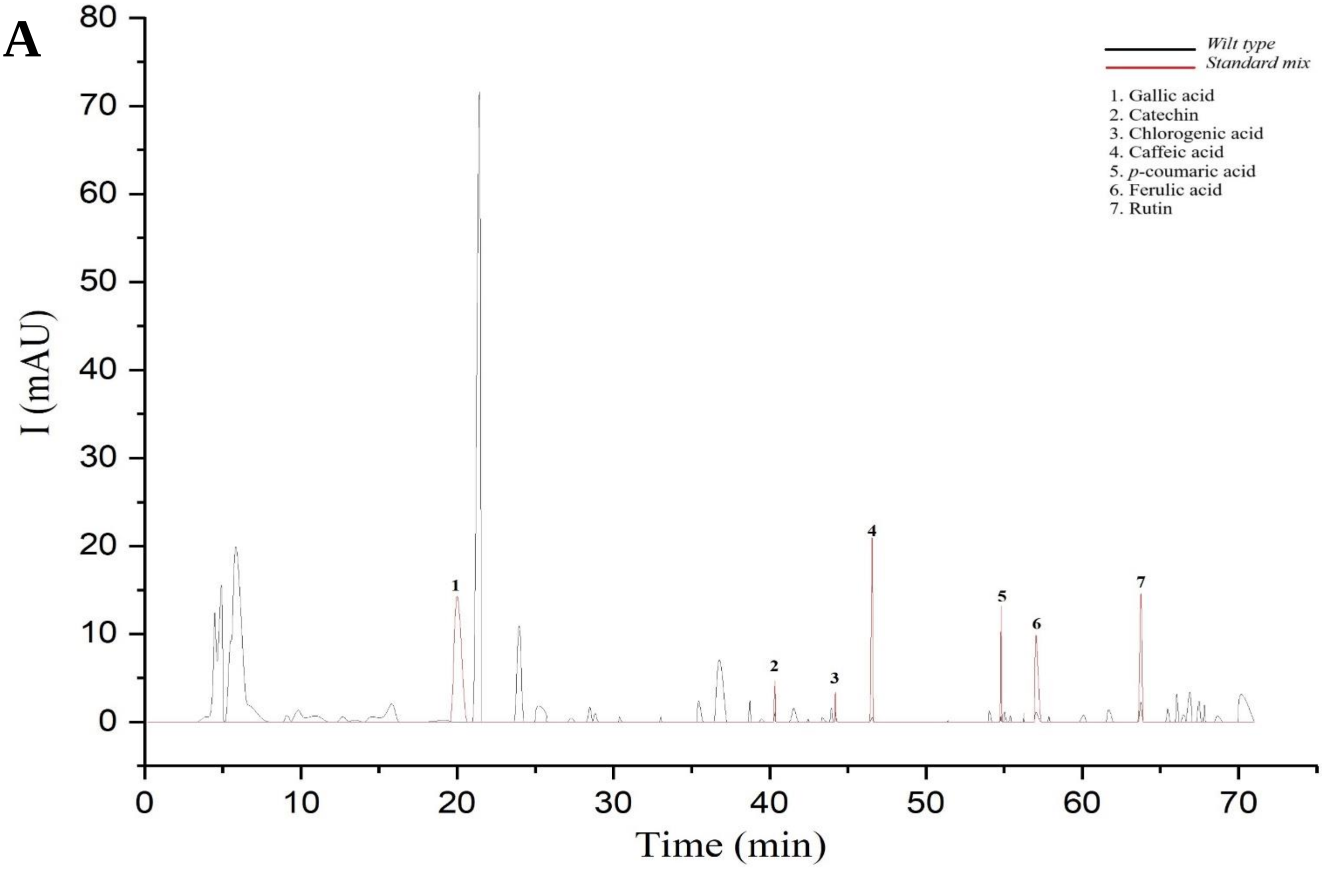
A
I (mAU)
Time (min)
Wilt type
Standard mix
1. Gallic acid
2. Catechin
3. Chlorogenic acid
4. Caffeic acid
5. p-coumaric acid
6. Ferulic acid
7. Rutin

B

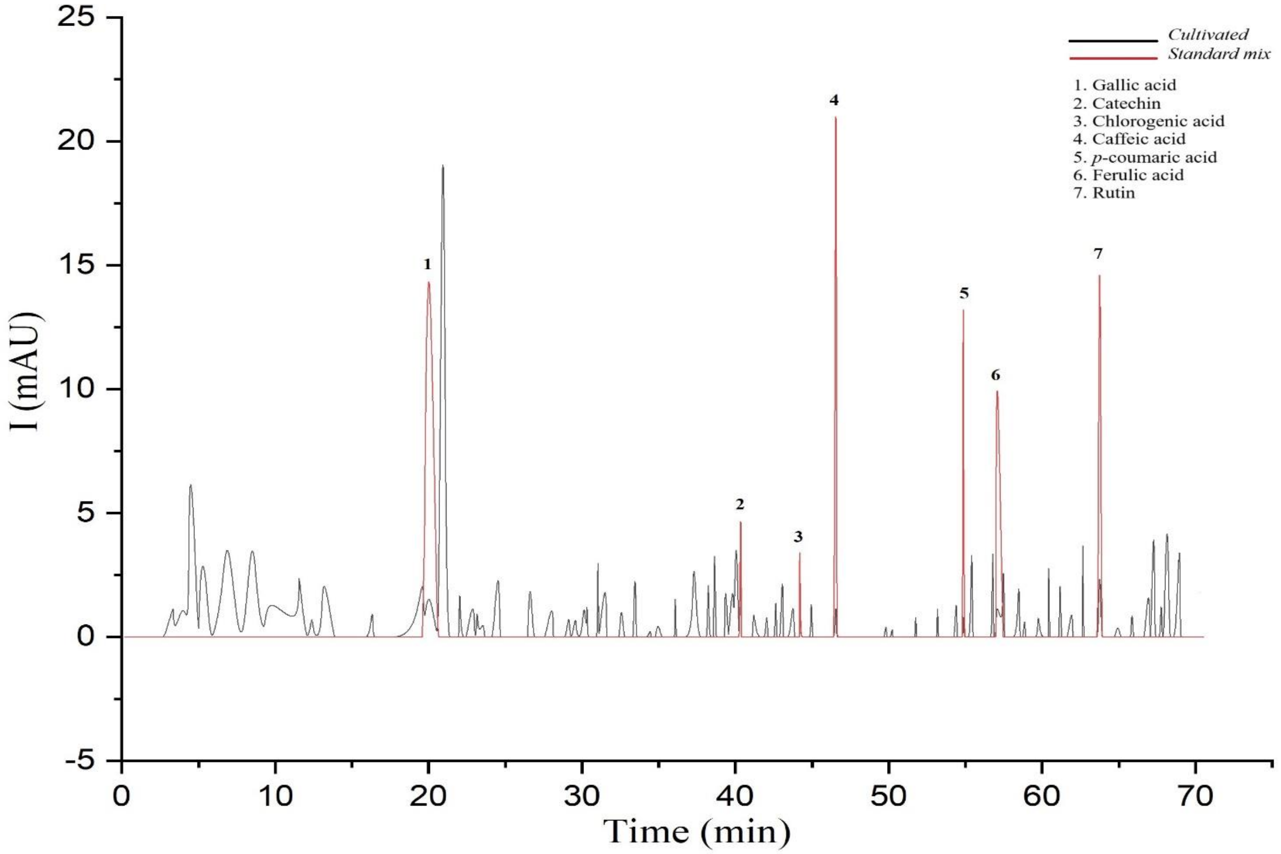

Cultivated
Standard mix
1. Gallic acid
2. Catechin
3. Chlorogenic acid
4. Caffeic acid
5. p-coumaric acid
6. Ferulic acid
7. Rutin
I (mAU)
Time (min)

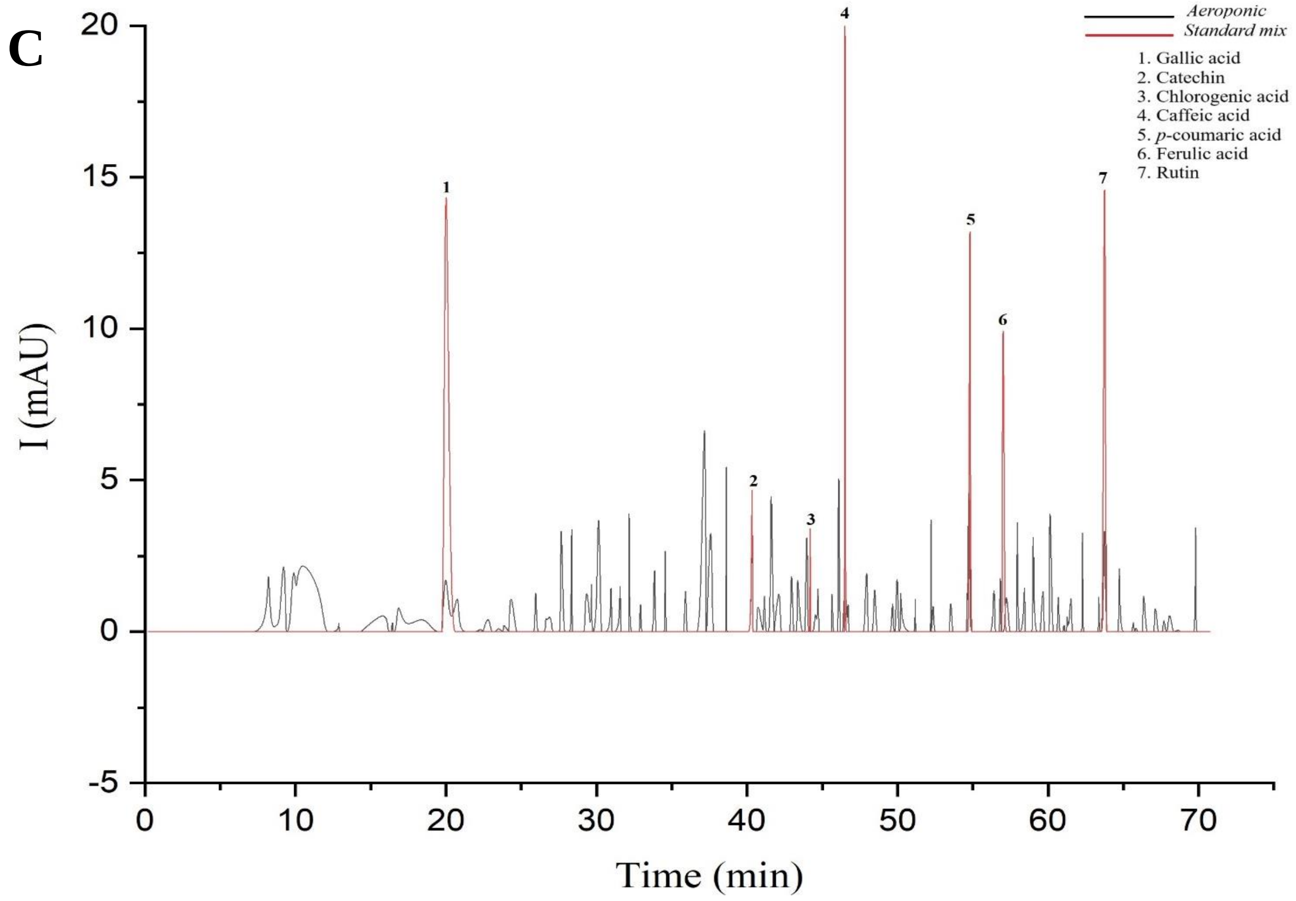

Figure S1. Chromatograms of raspberry extracts in ethanol. A: wild type, B: cultivated, C: aeroponic. All chromatograms were reported at a wavelength of 280 nm and overlapped with a standard mixture. Phenolics were qualified and quantified at different wavelengths, with respect to their maximum absorbance wavelengths. 1. gallic acid at 270 nm; 2. caffeic acid at 273 nm; 3. catechin at 280 nm; 4. *p*-coumaric acid at 310 nm; 5. ferulic acid at 325 nm; 6. chlorogenic acid at 327 nm, 7. rutin at 360 nm.

	Calibration range: 0.001 - 1 mg/mL
**Standard**	**Calibration curve**
Caffeic acid	$y=1.854473e^{-5} + 35.77557$; $R^2= 0.982$
Chlorogenic acid	$y=3.601749e^{-5} + 65.25935$; $R^2= 0.993$
Ferulic acid	$y=2.2515792e^{-5} + 36.49947$; $R^2= 0.990$
Gallic acid	$y=3.316615e^{-5} + 56.08516$; $R^2= 0.999$
p-Coumaric acid Catechin	$y=1.239896e^{-5} + 32.64989$; $R^2= 0.993$
Quercetin-3-O-rutinoside	$y=1.162342e^{-4} + 37.63891$; $R^2= 0.984$
	$y=8.602372e^{-5} + 65.9654$; $R^2= 0.999$

Figure S2. Calibration curve of phenolics used as external standards.
